# Value-impregnated factual claims and slippery-slope arguments

**DOI:** 10.1007/s11019-016-9723-4

**Published:** 2016-08-31

**Authors:** Gert Helgesson, Niels Lynøe, Niklas Juth

**Affiliations:** Stockholm Centre for Healthcare Ethics, Department of Learning, Informatics, Management and Ethics, Karolinska Institutet, 171 77 Stockholm, Sweden

**Keywords:** Slippery-slope arguments, Physician-assisted suicide, Hastening death, Value-impregnated estimations

## Abstract

Slippery-slope arguments typically question a course of action by estimating that it will end in misery once the first unfortunate step is taken. Previous studies indicate that estimations of the long-term consequences of certain debated actions, such as legalizing physician-assisted suicide, may be strongly influenced by tacit personal values. In this paper, we suggest that to the extent that slippery-slope arguments rest on estimations of future events, they may be mere rationalizations of personal values. This might explain why there are proponents even for strikingly poor slippery-slope arguments.

The main point of this paper is to connect two different philosophical ideas, namely those of slippery-slope argumentation and value-impregnated factual claims. We first give a brief presentation of each and then suggest, backed up by some empirical input, that slippery-slope arguments may rest on value-impregnated factual claims. We suggest that the less reasonable the slippery-slope arguments, the stronger they may be influenced by tacit personal values.

## Slippery-slope arguments

Slippery-slope arguments are usually based on estimations of what would happen in the future if a certain course of action were to be allowed. They claim that opening the door to that particular course of action will through a highly likely series of events lead to an outcome that is far removed from the intended outcome of the first step taken. This kind of argument is commonly used when someone wants to argue against a course of action (den Hartogh 2010; LaFollette 2005; Launis 2002, Launis 2010). Then opening the door to that particular course of action will eventually lead to a negative, if not catastrophic, outcome—hence the move downwards on the slippery slope. Therefore the door should never be opened.

A typical example of a slippery-slope argument in the medicoethical context is the argument against legalizing physician-assisted suicide (PAS) at the end of life. This is basically how it goes: If PAS is permitted, then one can expect there to be a continuous change in the application of criteria for eligibility, because of a slow change in the moral standards of society influenced by each of the steps taken. The consequences might be that more and more patients will become eligible for PAS, increasingly so for other reasons than what is in their best interest—e.g. not solely at the end of life or on the patient’s request. This development will sooner or later end up in a situation where atrocities in the application of PAS become common. (Mishara and Weisstub 2013; Shariff 2012; Rietjens et al. 2009) As a reaction to the degenerated practice, this will also significantly decrease the general population’s trust in the healthcare system, which will have further negative effects on health (Juth and Lynøe 2010; Helgesson et al. 2009). From this the argument concludes that we should avoid legalizing PAS. Similar arguments can be seen in discussions of sedation-therapy and intentionally hastening the death of imminently dying patients (Rydvall et al. 2014; Verhagen 2013; Camporesi and Boniolo 2008). The estimation of what will happen with the general public’s trust in healthcare is, although not the core issue, a common component in these slippery-slope arguments.

If a slippery-slope argument is based on estimations of future events, then determining to what extent it is likely that the estimation will come true is an empirical concern. Sometimes empirical studies can be carried out in order to examine the evidence for the estimations, while at other times this is not possible, for instance if there are no comparable situations from which to collect data. In the latter cases, the argument becomes irrefutable (Camporesi and Boniolo 2008).

## Value-impregnated factual claims

In a philosophy of science context, observations are often said to be hypothesis-impregnated or theory-impregnated, which means that theoretical assumptions and convictions influence observations (Rydvall et al. 2014). In analogy with theory-impregnated observations, we might speak of value-impregnated factual claims and judgments. A value-impregnated factual judgement is a judgement about fact(s) that is influenced by values. The general idea is that a factual judgement is value-impregnated if the person making it would not have made it, or would not have stressed its relevance to the same degree, if the person was not influenced by her values.

Note that the idea about value-impregnation of factual claims is distinct from ideas about value-ladenness and thick normative concepts. In the case of value-laden terms and thick normative concepts, the idea is that they have a descriptive content and in addition to this are loaded will value. For instance, it would be odd to say “He is honest/kind/forgiving, but I don’t thereby mean to say that there is anything good about him”, since honesty, kindness, and being forgiving, apart from having a descriptive content, designates something good. The point of arguing that some factual claims are value-impregnated is *not* to insist that they are loaded with value. Value-impregnation of factual claims concerns the explanation of *why* they are made. The explanation is that values influence what factual judgments are made (or how strongly they are stressed or defended), analogous to how theories can impinge on the perception and thereby influence what observations are made.

In contrast to theory-impregnated observations where (1) the theoretical influence on the observation can be said to be a precondition for making meaningful observations and (2) it is arguably the case that theory-impregnation of observations is unavoidable, value-impregnation of *factual claims* is avoidable and distorts/disturbs the factual judgement. This is not to deny that all *decisions* about what to do have an evaluative component: such a decision cannot be based on factual claims alone. However, also our factual claims may be affected by our value judgements, so that we, for instance, believe that something is the case because we want it to be the case or find it desirable that it would be the case. If we are not aware of the possibility of such value-impregnated factual judgements, we may think that we are more objective (i.e., to a larger extent steered by evidence for factual judgements, rather than by private values) than we actually are.

The case of particular interest in the healthcare context is value-impregnation stemming from personal values that disagree with official healthcare values. We suggest that value-impregnation of factual claims and judgements may be unintentional and unconscious, as well as conscious and intentional, in analogy with the potential influence of theoretical assumptions and convictions on observation.

An example of what would constitute value-impregnated factual judgements in the healthcare setting is exaggerated claims about the risk of abortion, or some other procedure, if the physician/nurse personally disapproves of the procedure or the frequent use thereof.

If the official healthcare values, as expressed in legislation and regulations, or otherwise stressed in the healthcare organization, run counter to those of the individual healthcare professional—especially if the latter are strongly held values and convictions—then it can be expected that these personal values, at least sometimes, will continue to influence the healthcare professional’s perception and behaviour, but if not officially accepted the influence is likely to continue in ways that are less easily detected. Rather than stating the values openly, the professional is more likely to let them affect the outcome by influencing what factual claims are on the table. Again, this move from the overt to the hidden is not necessarily intentional or conscious. The professional may even believe that he or she is acting in accordance with the official values.

## Slippery-slope arguments as value-impregnated factual claims

What we would now like to suggest is that slippery-slope arguments sometimes rest heavily on value-impregnated factual judgements. That is to say, not only can the factual judgement–normative judgement relation in this context be such that a normative conclusion is drawn *from* the conviction that things will develop in a certain way (slippery-slope style) if a certain decision is made and an evaluation of those outcomes. It can also go in the opposite direction: *from* the conviction that something should not be allowed (because it is considered bad), one’s factual judgements may be influenced by this conviction, for instance, into believing that there will be a slippery slope leading to disaster if a certain decision is made. This, of course, leads back to the normative conclusion, in a circular fashion.

A common feature of slippery-slope arguments, we take it, is that they, for good reasons, have little bite on those who are not already convinced of the fatality of the action at hand and require some rational grounds for that fear. In particular, the unavoidability of running through the entire purported series of events once the first step is taken in many cases seems to be gravely overstated—to the contrary, it seems both possible and feasible to come to a halt at each of the consecutive steps indicated if desirable (admittedly, whether it really is has to do with the case at hand). It seems that slippery-slope arguments are quite often used when a party in the debate is running out of good arguments for their position. This fits well with the “values first, factual judgements last” style of value-impregnated factual claims. To the extent that slippery-slope arguments should be understood in this vein, they mainly serve as a rationalization of already held value convictions and normative convictions.

## Lessons from empirical studies

Some recent studies provide empirical input for the discussion of value-impregnated factual claims and slippery-slope argumentation (Juth and Lynøe 2010; Rydvall et al. 2014; Björk et al. 2016; Lindblad et al. 2009).

In one of these studies, we examined a random sample of Swedish physicians about their attitudes towards physician-assisted suicide (PAS), and in another study we explored Swedish physicians’ attitudes towards intentionally hastening death (IHD) for imminently dying patients (Juth and Lynöe 2010; Rydvall et al. 2014). Apart from issues about their attitudes, we also asked the physicians what would happen with their own trust in healthcare if these practices were legalised. On average across the two studies, 61.2 % (n = 627) stated that their own trust would decrease, 12.2 % (n = 125) stated that it would increase, and 26.6 % (n = 273) stated that it would not influence their own trust in healthcare.

We made the assumption that there is an association between judging the legalisation of PAS/IHD as a bad thing, since it would make these actions legal under specified conditions, and estimating that one’s own trust in healthcare would decrease if such legislation were put in place. Similarly, we assumed that if legalisation in favour of the two practices was considered a good thing, the physician would estimate that their own trust in healthcare would increase if these legal changes were made. Finally, we assumed that those who found legalizing PAS/IHD as neither good nor bad would state that their own trust in healthcare would not be influenced by a change in legislation. If our assumption is reasonable, it seems clear that a majority implied that legalizing PAS and IHD would be a bad thing.

In order to study whether or not there was a correlation between the physicians’ statements about what would happen with their own trust in healthcare and their estimations of what would happen with the general public’s trust in healthcare if the two practices were legalised, we also provided these questions with the same response options in both studies. We found a good interrater agreement for PAS [weighed Kappa: 0.695 (95 %CI 0.640–0.751)] and a rather good interrater agreement for IHD [(0.585 (95 %CI 0.492–0.678)]; the average weighed Kappa for both practices was good (0.620). Among those whose own trust would decrease (n = 627), we found that 83.4 % also estimated that the general public’s trust would decrease (Juth and Lynöe 2010; Rydvall et al. 2014). It is worth noting that while physicians’ decreased trust in healthcare may not be expected to have any impact on patient safety, patients’ lack of trust in the healthcare system may indeed have a negative impact on health outcomes, since their lack of trust may change their behaviour towards healthcare staff and regarding their treatment recommendations in unfavourable ways.

It should further be noted that another empirical study, carried out among the general population, has indicated that legalizing PAS would not jeopardise the general public’s trust in healthcare—rather, trust was estimated not to be influenced or to increase (Lindblad et al. 2009).

We interpret these findings as an indication that the physicians’ personal values regarding PAS and IHD tend to affect their estimations of what would happen with the general public’s trust in healthcare if PAS and IHD were legalised. These factual estimations appear to be independent of the respondents’ personal attitudes towards PAS and IHD, while they most likely are not. And, as we saw above, estimations of effects on the general public’s trust in healthcare are usually important components in slippery-slope arguments regarding PAS—and also regarding IHD.

If this interpretation is correct, one may wonder why physicians make value-impregnated factual claims, for instance, instead of simply stating their values and attitudes regarding these practices. One potential influencing factor is that physicians are expected to act in a rational, reasonable way based on empirical evidence and the generally accepted goals of healthcare. Acting based on personal values is understood as inappropriate in Swedish healthcare, which does not leave any room for conscientious objections (Svennerlind 2009). If physicians nevertheless want to let their personal values influence the outcome, slippery-slope arguments might come in handy since they can give the impression of being based only on factual judgements and some shared dislike for the catastrophic outcome. At the same time they conceal what is really going on.

We are not suggesting in this paper that all slippery-slope arguments are of the same kind in all important respects. Thus, while value-impregnated factual claims may be involved in some instances, they may not be so in others. However, we suggest that in the healthcare context, when slippery-slope arguments occur, it might be worthwhile to look out for value-impregnated factual claims.

## Conclusion

The heart of slippery-slope reasoning is to claim that a certain course of action should not be adopted or allowed, because once we have taken the first step the trajectory is set, and we are to face very negative consequences. Hence, slippery slope arguments make the estimation that certain undesirable future events are unavoidable, or at least highly likely, once the first unfortunate step on that path is taken. In this paper, we have suggested that such factual estimations in slippery-slope arguments may be value-impregnated. That is, the estimations are made because the persons making them have a certain attitude to the action at hand. There is some support that estimations among Swedish physicians regarding the general public’s trust in healthcare as a consequence of certain debated reforms, such as legalizing PAS, take this form.

If we are to take slippery-slope arguments seriously, they must contain reasonable estimations of future events based on carefully conducted empirical investigations, or at least on good theoretical reasons why a certain development is likely to take place. Otherwise, there is a considerable risk that the estimation of the consequences of a certain decision or act is merely a rationalization impregnated by the person’s own attitudes or values.

## References

[CR1] Björk, J., Lynöe, N., Juth, N. Empirical and philosophical analysis of physicians’ judgments of medical indications. *Clinical Ethics*. Accepted. 10.1177/1477750916657666 PMC5117119 27904437

[CR2] Camporesi S, Boniolo G (2008). Fearing a non-existing Minotaur? The ethical challenges of research on cytoplasmic hybrid embryos. Journal of Medical Ethics.

[CR3] den Hartogh, G. 2010. The Slippery Slope Argument. In *A Companion to Bioethics*, 321–332. Wiley-Blackwell.

[CR4] Helgesson G, Lindblad A, Thulesius H, Lynøe N (2009). Reasoning about physician-assisted suicide: analysis of comments by physicians and the Swedish general public. Clinical Ethics.

[CR5] Juth N, Lynøe N (2010). Do strong values influence estimations of future events?. Journal of Medical Ethics.

[CR6] LaFollette H (2005). Living on a slippery slope. Journal of Ethics.

[CR7] Launis V (2002). Human gene therapy and the slippery slope argument. Medicine, Health Care and Philosophy.

[CR8] Launis V (2010). Cosmetic neurology: Sliding down the slippery slope?. Cambridge Quarterly of Healthcare Ethics.

[CR9] Lindblad A, Löfmark R, Lynøe N (2009). Would physician-assisted suicide jeopardize trust in medical services? An empirical study of attitudes among the general public in Sweden. Scandinavian Journal of Public Health.

[CR10] Mishara BL, Weisstub DN (2013). Premises and evidence in the rhetoric of assisted suicide and euthanasia. International Journal of Law and Psychiatry.

[CR11] Rietjens J, Rietjens AC, van der Maas PJ (2009). Two decades of research on Euthanasia from the Netherlands. What have we learnt and what questions Remain?. Bioethical Inquiry.

[CR12] Rydvall A, Juth N, Sandlund M, Lynøe N (2014). Are physicians’ estimations of future events value-impregnated? Empirical study of double intentions when providing treatment that shorten a dying patient’s life. Medicine, Health Care and Philosophy.

[CR13] Svennerlind, C. 2009. Tillräknelighet i svensk rätt. (Accountability in Swedish Law.) In *Tillräknelighet* (Accountability). eds. Radovic, S., Anckarsäter, H., 51–106. Lund:Studentlitteratur.

[CR14] Shariff MJ (2012). Assisted death and the slippery slope-finding clarity amid advocacy, convergence, and complexity. Current Oncology.

[CR15] Verhagen AA (2013). The Groningen Protocol for newborn euthanasia; which way did the slippery slope tilt?. Journal of Medical Ethics.

